# Twitter users’ reaction to a chain pharmacy’s decision to end tobacco sales

**DOI:** 10.1186/s12971-015-0060-9

**Published:** 2015-11-04

**Authors:** Patricia A. McDaniel, Hannah Patzke, Ruth E. Malone

**Affiliations:** Department of Social and Behavioral Sciences, School of Nursing, University of California, San Francisco, 3333 California Street, Ste. 455, San Francisco, CA 94118 USA

## Abstract

**Background:**

Reducing the number of tobacco outlets may help reduce smoking uptake and use; public support for such action is essential. We explored how Twitter users responded to the announcement by US pharmacy chain CVS that it was voluntarily ending tobacco sales.

**Methods:**

We used Twitter’s application programming interface to retrieve tweets and retweets posted over an 8-day period in February 2014 that contained two trending CVS-related hashtags (#cvs and #cvsquits). We manually coded 6,257 tweets as positive, negative, or neutral.

**Results:**

The majority of tweets were positive (56.0 %) or neutral (39.4 %).

**Conclusions:**

There was little disapproval of CVS’s decision to end tobacco sales among Twitter users, possibly due to the voluntary nature of the decision. The level of support suggests that CVS’s image and bottom line will not suffer as a result. Further voluntary actions to end tobacco sales – which may lay the groundwork for legislation -- should be incentivized and supported.

Recently, the U.S. Surgeon General’s Report identified tobacco sales restrictions as a promising “endgame” strategy for the tobacco epidemic ([1], p. 858). Such restrictions could include reducing the number of tobacco outlets. Tobacco outlet density increases the likelihood of smoking among minors and adults [2–5], and living in close proximity to tobacco outlets is associated with unsuccessful quit attempts [6, 7].

As many pharmacists consider the sale of tobacco unethical, eliminating pharmacy tobacco sales may be an obvious starting point for tobacco outlet reduction [8–12]. Some countries, including Australia and New Zealand, prohibit pharmacy tobacco sales. In the U.S., a handful of cities do so [13]; however, some pharmacies have voluntarily ended tobacco sales. Independent pharmacies were among the first [8, 14–16], followed by some larger chains. Target was the first national chain to voluntarily end tobacco sales in 1996; Wegmans, a regional grocery and pharmacy chain, did so in 2008 [17]. The most recent national pharmacy chain to voluntarily end tobacco sales was CVS Caremark (now CVS Health) [18]. It did so, in part, because of a changing pharmacy landscape. CVS (and other pharmacies) are seeking to become a more significant part of the healthcare system through the introduction of retail health clinics; CVS regarded tobacco sales as inconsistent with that goal [18].

In announcing its decision, CVS relied on traditional media and on social networking sites, including Twitter. Twitter is a microblogging website launched in 2006 whose users read and write short messages or “tweets” (up to 140 characters) on a variety of topics; the site also allows users to send public messages to one another and create a network of “followers” who subscribe to their tweets. As of January 2014, 19 % of all American adult internet users use Twitter, with use significantly higher among men, younger age groups (18–29 years), and lower income groups [19]. Worldwide, 316 million users send 500 million tweets per day [20]. Twitter’s speed of information delivery and ability to reach a wide audience make it an attractive tool for organizations to reach target audiences; it is also an important source of opinion data. In some instances, public sentiment on Twitter averaged over several days closely matches that of a traditional national telephone survey [21]. Twitter activity prior to elections has also been found to reflect the political sentiments of the electorate, with the number of tweets mentioning a political party corresponding to each party’s vote share [22].

In this paper, we explore how Twitter users responded to CVS’s announcement that it had decided to end tobacco sales and discuss the implications for efforts to reduce tobacco’s retail availability.

## Methods

We used Twitter’s publicly available application programming interface (API) to retrieve tweets containing the hashtags #cvsquits and #cvs sent February 5, 2014 (the day CVS announced its decision) to February 12, 2014. (Because we performed our search prior to March 2014, when Twitter offered researchers access to all tweets on record [23], we only had access to 1 % of the population of tweets through the Twitter API.) [23, 24] Hashtags are short keywords prefixed by the hash symbol (#); they identify and group conversations among all Twitter users on a particular topic, including users who may not be connected to one another via existing networks.

We chose #cvsquits because it was the official announcement hashtag, and #cvs because it was a clear alternative used by many to weigh in on the trending discussion. We also noted that both hashtags were “trending” during the week after CVS made its announcement. Twitter determines which topics are trending and lists them throughout the site [25]. According to the company:

Twitter Trends are automatically generated by an algorithm that attempts to identify topics that are being talked about more right now than they were previously. The Trends list is designed to help people discover the “most breaking” breaking news from across the world, in real-time. The Trends list captures the hottest emerging topics, not just what’s most popular [25].

We also conducted searches for other potentially relevant hashtags (e.g., #cigarettes and #tobacco), including negative hashtags (e.g., #cvsfail and #cvssucks), but each retrieved fewer than 20 tweets.

We entered #cvs and #cvsquits into Twitter’s API and copied all of all of the publically available tweets that we retrieved into an Excel spreadsheet. (More information on how to use Twitter’s API to search for tweets can be found on the Twitter website) [26]. We retrieved 8,645 tweets, 2,761 for #cvsquits, and 5,884 for #cvs. We excluded 2,388 tweets we classified as spam (trending hashtags used to link to another subject), unrelated (e.g., #cvs tweets that referred to coupons), in a language other than English, or duplicates (e.g., both #cvs and #cvsquits used in the same tweet), leaving us with 6,257 tweets (including re-tweets). Re-tweets occur when a Twitter user copies another user’s message (referencing the original author) to further comment on the issue or to post the information to her/his own followers [27]. Because they are not equivalent to duplicates, we chose to include re-tweets.

The second author coded all tweets as positive (reflecting support for the decision, e.g., “#CVS, I’m proud of you! (Corporate Values Showing)”), negative (objecting to or raising concerns about the decision, e.g., “CVS now on the slippery slope #CVS”), or neutral (simply relaying the news, e.g., “#CVS stops selling cigarettes”). In cases where the opinion was unclear and the tweet included a link to a news or other article, the coder read that article to determine how to classify the tweet. She referred questionable tweets to the first author for review and resolution. The first author also coded a random sample of 1 % of all tweets (*n* = 63) to check agreement; the two coders assigned the same codes 90 % of the time.

## Results

The majority of tweets (74.4 %) were sent the day of CVS’s announcement, with the number declining as the week progressed. Only 4.6 % of tweets expressed a negative view of CVS’s decision to end tobacco sales; the majority expressed a positive (56.0 %) or neutral (39.4 %) view.

## Discussion

Our study has limitations. Because the Twitter website did not indicate how the tweets that were accessible through its API were chosen, they may not have represented a random sample. Thus, our results may be biased in unknown ways. Moreover, the applicability of our findings to public opinion more generally is unclear, as research supporting a link between public sentiment on Twitter and public opinion more broadly is now five years old [21, 22], a long period of time in the fast-changing world of social media. A strength of our study is our reliance on manual rather than automated coding to classify tweet opinion. The accuracy of automated coding can be poor, in part because human coders can more easily detect sarcasm and understand slang [24, 28].

CVS’s decision to end tobacco sales was viewed mostly positively, or in a neutral fashion by those who chose to tweet about it. The small number of negative tweets suggests that there was little disapproval among Twitter users. This may be explained, in part, by the minimal impact of the decision on tobacco’s retail availability: overall, pharmacies reportedly account for approximately 3.5-4 % of all tobacco sales [29, 30]. Twitter users’ reactions may also be explained by the voluntary nature of the decision. Legislation prohibiting tobacco sales in pharmacies may have provoked more controversy; indeed, nationally, only 31.3 % of Americans support a ban on pharmacy tobacco sales [31]. While the public may be more inclined to support voluntary policies, voluntary actions may nonetheless lay the groundwork for public policies, by familiarizing the public and policymakers with the concept of restricting tobacco sales to particular retail outlets, and by further denormalizing such sales.

## Conclusion

The level of support for (or indifference to) CVS’s decision to end tobacco sales suggests that CVS’s image and bottom line will not suffer as a result. Indeed, approximately one year after ending tobacco sales, CVS announced that its profits had increased 2.1 % [32]. Given that some chain pharmacies may be less well-positioned financially to end tobacco sales [29] such actions should be incentivized (e.g., through tax breaks for participating businesses) and supported, enhancing public health efforts to reduce the ubiquitous availability of tobacco in retail stores.
